# Two-dimensional silicon bismotide (SiBi) monolayer with a honeycomb-like lattice: first-principles study of tuning the electronic properties

**DOI:** 10.1039/d0ra05026a

**Published:** 2020-09-02

**Authors:** Asadollah Bafekry, Fazel Shojaei, Mohammed M. Obeid, Mitra Ghergherehchi, C. Nguyen, Mohammad Oskouian

**Affiliations:** Department of Physics, University of Guilan 41335-1914 Rasht Iran Bafekry.asad@gmail.com; Department of Physics, University of Antwerp Groenenborgerlaan 171 B-2020 Antwerp Belgium; Department of Chemistry, Faculty of Sciences, Persian Gulf University Bushehr 75169 Iran; Department of Ceramics, College of Materials Engineering, University of Babylon Hilla 51002 Iraq; Institute of Research and Development, Duy Tan University Da Nang 550000 Vietnam; College of Electronic and Electrical Engineering, Sungkyunkwan University Suwon Korea mitragh@skku.edu

## Abstract

Using density functional theory, we investigate a novel two-dimensional silicon bismotide (SiBi) that has a layered GaSe-like crystal structure. *Ab initio* molecular dynamic simulations and phonon dispersion calculations suggest its good thermal and dynamical stability. The SiBi monolayer is a semiconductor with a narrow indirect bandgap of 0.4 eV. Our results show that the indirect bandgap decreases as the number of layers increases, and when the number of layers is more than six layers, direct-to-indirect bandgap switching occurs. The SiBi bilayer is found to be very sensitive to an E-field. The bandgap monotonically decreases in response to uniaxial and biaxial compressive strain, and reaches 0.2 eV at 5%, while at 6%, the semiconductor becomes a metal. For both uniaxial and biaxial tensile strains, the material remains a semiconductor and indirect-to-direct bandgap transition occurs at a strain of 3%. Compared to a SiBi monolayer with a layer thickness of 4.89 Å, the bandgap decreases with either increasing or decreasing layer thickness, and at a thicknesses of 4.59 to 5.01 Å, the semiconductor-to-metal transition happens. In addition, under pressure, the semiconducting character of the SiBi bilayer with a 0.25 eV direct bandgap is preserved. Our results demonstrate that the SiBi nanosheet is a promising candidate for designing high-speed low-dissipation devices.

## Abstract 2

The modulation of the electronic properties of SiBi monolayer *via* external means, including layer thickness, electric field and mechanical strain are explored with DFT method.

## Graphical abstract

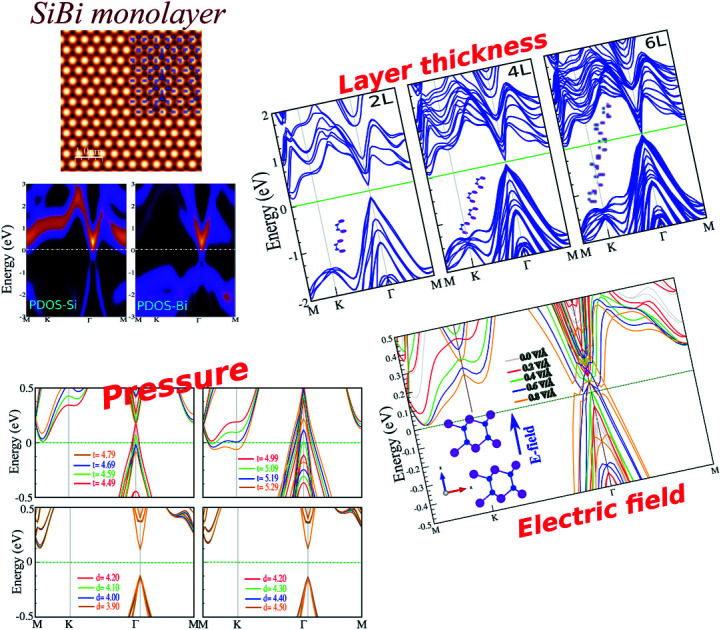

## 1 Introduction

The successful isolation of graphene in 2004 and studies of its amazing physical properties,^1^ have sparked tremendous research interest in searching for other new members of the fast growing two-dimensional materials (2DMs) family. The majority of 2DMs so far identified are multi-element compounds. However, a few 2DMs have either been theoretically or experimentally identified to exist in the elemental form, and they belong to the main groups of IIIA (B, Al, Ga, In), IVA (C, Si, Ge, Sn, Pb), VA (P, As, Sb, Bi), and VIA (Te, Se).^2,3^ The electronic properties of this class of 2DMs differ significantly: the borophene monolayer with anisotropic buckling shows metallic characteristics;^4^ silicene^5^ and germanene^6^ monolayers with a buckled honeycomb lattice are semimetals with Dirac cones similar to graphene; black phosphorene is a semiconductor with a thickness-tunable direct band gap, high carrier mobilities, and high in-plane anisotropy;^7,8^ and a bismuthene monolayer has also been predicted to show topological insulating behavior at room temperature.^9^ Apparently, a much greater degree of flexibility in tuning the electronic structure of 2DMs can also be achieved by combining two or more types of elements with different properties. A large number of possible 2DMs are binary compounds of main group elements. According to the literature, to date, main group binary 2DMs with combinations of IIIA–IVA (*e.g.*, B_4_4C_3_, Al_*x*_C),^10,11^ IIIA–VA (*e.g.*, BN, BP),^12^ IIIA–VIA (*e.g.*, GaS, GaSe),^13–16^ IVA–VA (*e.g.*, GeP, GeAs),^17–20^ IVA–VIA (*e.g.*, SnS, SnSe)^21,22^ and VA–VIA (*e.g.*, As_2_S_3_, As_2_Se_3_)^22,23^ have been already identified.

Just recently, 2D IVA–VA binary semiconductors with chemical compositions of IVV (GeP, GeAs, SiP, SiAs),^24,25^ IVV2, (SiP_2_, SiAs_2_, GeAs_2_)^26,27^ IVV3 (GeP_3_, SnP_3_),^28^ and IVV5 (GeP5),^29^ have gained considerable research attention after little consideration since their first synthesis. Several experimental and theoretical studies have investigated the exfoliation,^17–20,30,31^ band gap,^32,33^ electrical transport,^19,20,34^ thermal conductivity,^32^ and photocatalytic activity for water splitting reactions^33,35^ of these 2DMs. Similar to their 2D elemental parents, it has been theoretically predicted that 2D IVV, IVV2, and IVV3 compounds also possess 2D polymorphs with different lattices. As an example, calculations proposed a meta-stable GaS-like structure with *P*6*m*2 space group for IVV compounds, which is slightly less stable than the experimentally observed 2D low symmetry monoclinic phase.^36–39^ Calculations show that GaS-like IVV monolayers are all semiconductors except for CBi and PbN, which exhibit metallic behavior.^36^ These 2D polymorphs can exhibit quite different electronic properties. Therefore, such rich structural diversity further enhances the electronic properties of elemental 2DMs.

The ability to reversibly control the electronic properties of 2DMs, plays a key role in dictating their potential future applications. Modulating the band gap of 2DMs can be obtained by various external means: applications of different types of vertical and in-plane strains, application of an electric field, doping, surface/edge functionalization, varying thickness of the 2DM, and heterostructure formation, are a few examples.^40–44^

In this work, using density functional theory, we proposed a novel 2D silicon bismotide (SiBi) with a layered GaSe-like crystal structure that possesses a low indirect band gap of 0.65 eV, calculated using HSE06 with inclusion of the spin orbit coupling effect. *Ab initio* molecular dynamic simulations at 300 K and phonon dispersion calculations suggest its good thermal and dynamical stability. The modulation of electronic properties of the SiBi monolayer *via* external means, including layer thickness, electric field (E-field), and different types of in-plane and out-of-plane strain (pressure) have also been investigated using fully relativistic calculations. We found that the band gap value and even the nature of the band gap of the SiBi monolayer can be highly modulated by these external means.

## 2 Method

In this work, we report results of our DFT calculations for the electronic structure as implemented in the OpenMX 3.8 package.^45^ The Perdew–Burke–Ernzerhof approach from the generalized gradient approximation (PBE-GGA)^46^ is applied to describe the exchange-correlation functional and the norm-conserving pseudopotentials.^47^ The wave functions are obtained from the linear combination of multiple pseudoatomic orbitals (LCPAOs), which can be generated by a confinement scheme.^48,49^ The PAO basis functions were specified by s^2^p^2^d^1^ for Si atoms with the cutoff radii of the basis functions set to the value of seven. After convergence tests, we chose an energy cutoff of 400 Ry for the pristine SiBi monolayer. The atomic positions are optimized using a quasi-Newton algorithm for atomic force relaxation, where the structure was fully relaxed until the force acting on each atom was less than 1 meV Å^−1^. A *k*-point mesh of 23 × 23 × 1 of the Monkhorst–Pack^50^ is used to obtain both the atomic structure and electronic characteristics. In order to avoid all non-physical interactions between adjacent layers in the SiBi monolayer, a large vacuum layer of 20 Å is applied along the *z* direction. To get a clear picture of the van der Waals interactions, which dominate the layered SiBi monolayer, we used the empirical dispersion method of DFT-D2.^51^ The vibrational characteristics of the SiBi monolayer are obtained by performing the finite-displacement method within the PHONOPY code.^52^ Furthermore, we also provide the scanning tunneling microscopy (STM) simulations using the Tersoff–Hamann^53^ in WSxM package.^54^

## 3 SiBi monolayer

**Fig. 1:**
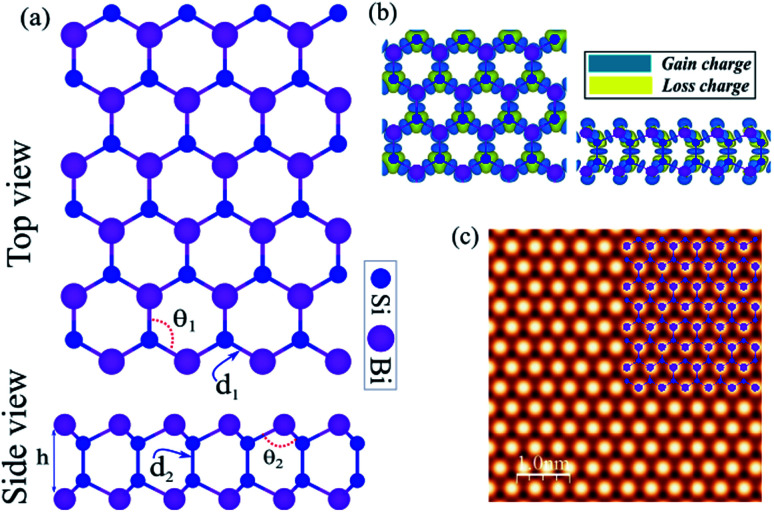
(a) Atomic structure of the SiBi monolayer with the primitive unit cell indicated by a red hexagonal. (b) Difference charge density. The blue and yellow regions represent the charge accumulation and depletion, respectively. (c) Simulated STM image of the SiBi monolayer. The inset structure represents the repeating unit cell.

The honeycomb atomic lattice of the SiBi monolayer exhibits the space group *P*3*m*1, as shown in Fig. 1(a). Notice that the crystal structures of SiBi consist of 2-Si layers sandwiched between Bi-layers in the order Bi–Si–Si–Bi. Before investigating the electronic properties, we first optimize the geometric structure with full optimization of all the atoms, and determine the crystal lattice parameters. We found that the lattice parameter is 4.09, while the bond lengths are *d*_1_ = 2.69 Å and *d*_2_ = 2.31 Å. In addition, the bond angles are calculated to be *θ*_1_ = 98.94 and *θ*_2_ = 118.63° and the thickness (Δ*z*) is determined to be 4.89 which is the distance between Bi–Bi atoms in the SiBi monolayer. The structural parameters are listed in Table 1. The difference in charge density of SiBi is shown in Fig. 1(b), where the blue and yellow regions represent the charge accumulation and depletion, respectively. The difference in charge density (Δ*ρ*) is defined as:

1 Δ*ρ* = *ρ*_tot_ − *ρ*_Si_ − *ρ*_Bi_

where *ρ*_tot_, *ρ*_Si_ and *ρ*_Bi_ represent the charge densities of the SiBi and isolated atoms, respectively. Notice that the negatively charged Bi atoms are surrounded by positively charged Si atoms, which indicates a charge transfer from Si to Bi atoms, resulting in each Bi atom gaining about 0.04*e* from the adjacent Si atom. The charge redistribution is due to the different electro-negativities of 1.9 (Si) and 2.02 (Bi). The simulated STM image of the SiBi monolayer is shown in Fig. 1(c), which overlays with its structure. It is easy to recognize and correlate them with the corresponding atomistic structure, also we can see that the Bi atoms are brighter than those of the Si atoms.

**Table 1:** The calculated lattice constant (*a*); the bonding lengths between Si and Bi (*d*_Si–Bi_), and Si and Si (*d*_Si–Si_); bonding angles between Bi–Si–Bi atoms *θ*_1_, and Bi–Si–Si *θ*_2_; thickness defined by the distance between the largest and smallest *z* coordinates of Bi atoms (*t*); charge transfer (Δ*Q*); and cohesive energy per atom (*E*_coh_), of the SiBi monolayer. The electronic states (ES) are specified as semiconductor (SC), band gaps within PBE (*E*^PBE^_g_), band gap within HSE (*E*^HSE^_g_), the band gap inside the parentheses takes SOC into consideration and is given in eV. VBM and CBM positions

	*a* (Å)	*d*_Si–Bi_ (Å)	*d*_Si–Si_ (Å)	*θ*_1_ (°)	*θ*_2_ (°)	*t* (Å)	Δ*Q* e	*E*_coh_ (eV per atom)	ES	*E* ^PBE^_g_ (eV)	*E* ^HSE^_g_ (eV)	VBM/CBM
SiBi	4.09	2.69	2.31	98.94	118.63	4.89	0.04	4.65	SC	1.1 (0.4)	1.3 (0.65)	*Γ*/*M*–*K*

The cohesive energy and the phonon spectrum of the SiBi monolayer are also recorded to confirm the structural and dynamical stability. The cohesive energy (*E*_coh_) per atom was calculated using the following equation:2
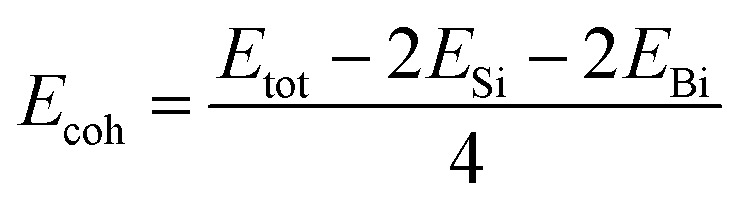
where *E*_tot_ represents the total energy of the SiBi monolayer; *E*_Si_ and *E*_Bi_ represent the energies of isolated single Si and Bi atoms, respectively. The cohesive energy of SiBi is calculated *E*_coh_ = 4.65 eV per atom and proves that SiBi may be stable.

**Fig. 2:**
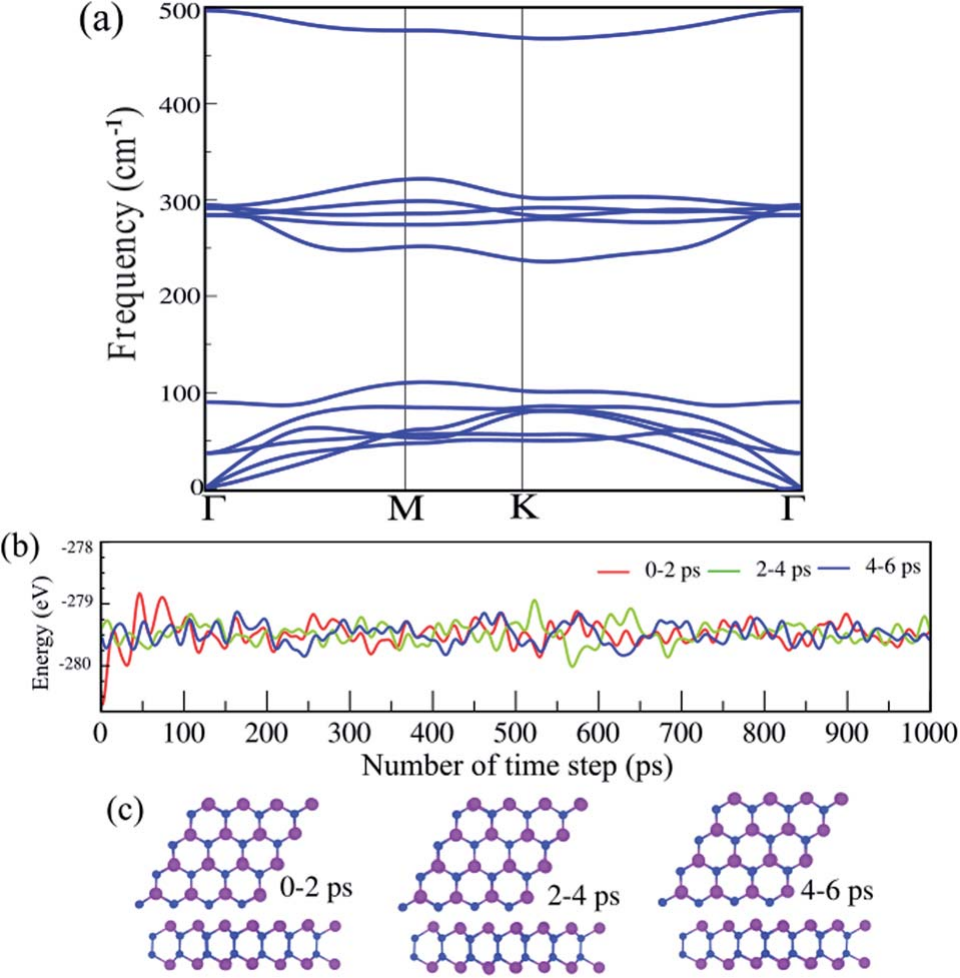
(a) Phonon dispersion spectra, (b) AIMD simulation, and (c) optimized structures of the SiBi monolayer.

The phonon dispersion spectra of the SiBi monolayer are displayed in Fig. 2(a). One can find that all the dispersion curves of the SiBi monolayer are positive with the linear acoustic branch along the *Γ* point, confirming the kinetically stable SiBi. The thermal simulations of the SiBi monolayer at room temperature, by performing the AIMD simulation, are depicted in Fig. 2(b). The time step was set to 2 fs (1000 steps) with a total simulation time of 6 ps. The structure snapshots are taken at the end of each simulation every 2 ps. Our AIMD simulation demonstrates that the atomic structure of SiBi is maintained at a room temperature of 300 K after heating to 6 ps, as illustrated in Fig. 2(c). Moreover, the difference in the total energy of SiBi before and after 6 ps is quite small. These demonstrations confirm that the SiBi is thermally stable at room temperature.

**Fig. 3:**
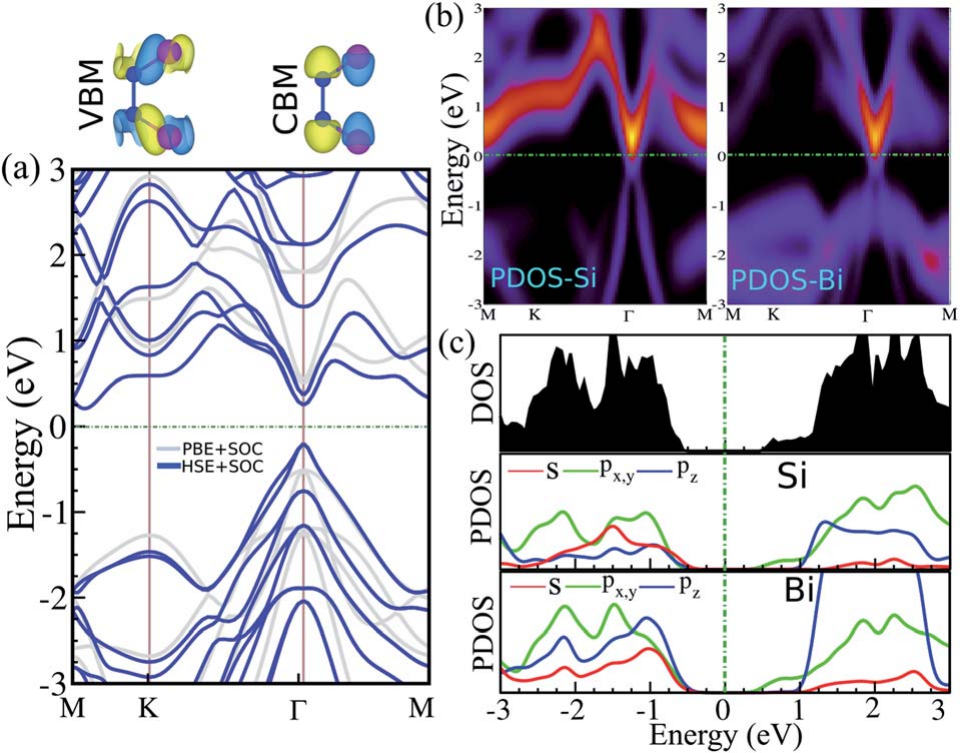
(a) Electronic band structure, (b) intensity map, (c) DOS and PDOS of the SiBi monolayer within PBE considering SOC. Band structure within PBE and HSE, indicated with the light gray line and the blue line, respectively. Charge densities of the conduction band minimum (CBM) and the valance band maximum (VBM) are shown at the top of the panel. The zero energy is set to the Fermi level.

The electronic band structure, intensity map, DOS and PDOS of the SiBi monolayer within PBE, considering SOC, are shown in Fig. 3(a–c), respectively. Our results on the electronic band structure within PBE, with (and without) considering SOC, show that the SiBi monolayer is a semiconductor with an indirect (direct) band gap of 0.4 (1) eV. In addition, the CBM of SiBi occurs along the *M*–*K* direction, and the VBM is located at the *Γ*-point. It can be seen that the HSE and HSE + SOC approach does not change the sort of indirect semiconducting character, and the band gaps were estimated to be 1.3 eV and 0.65 eV, respectively. The charge densities of conduction band minimum (CBM) and the valance band maximum (VBM) are shown in the top panel in Fig. 3(a). Notice that the electron effective mass along *Γ* → *K* (*M*) is 0.15 (0.29) 
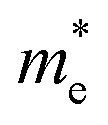
, while the hole effective mass is −0.1 and −0.16 
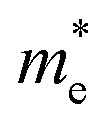
 along *Γ* → *K* and *Γ* → *M*, respectively. These light electron and hole effective masses lead to the high carrier mobility in the SiBi monolayer. The density and partial density of states (DOS and PDOS) are also considered to understand the contribution of all orbitals in SiBi. As shown in Fig. 3(c), the DOS of SiBi exhibit multiple van Hove singularities over the entire energy range, which is consistent with the 2D nature of a single-layer material. It is observed that the states near the Fermi-level have contributions from the p orbitals of Si and Bi. The contributions from the p_*z*_ orbital of Si and Bi are much higher than those from the p_*x*,*y*_ orbitals. The fact that the p_*z*_-orbitals are dominant is caused by the sp^3^-like bond of Si and the sp^2^-like bond of Bi forming the SiBi monolayer. Notice that, the states closest to the VBM and CBM of SiBi have contributions from the Bi-p and Si-p orbitals, respectively.

## 4 Layer thickness

**Fig. 4:**
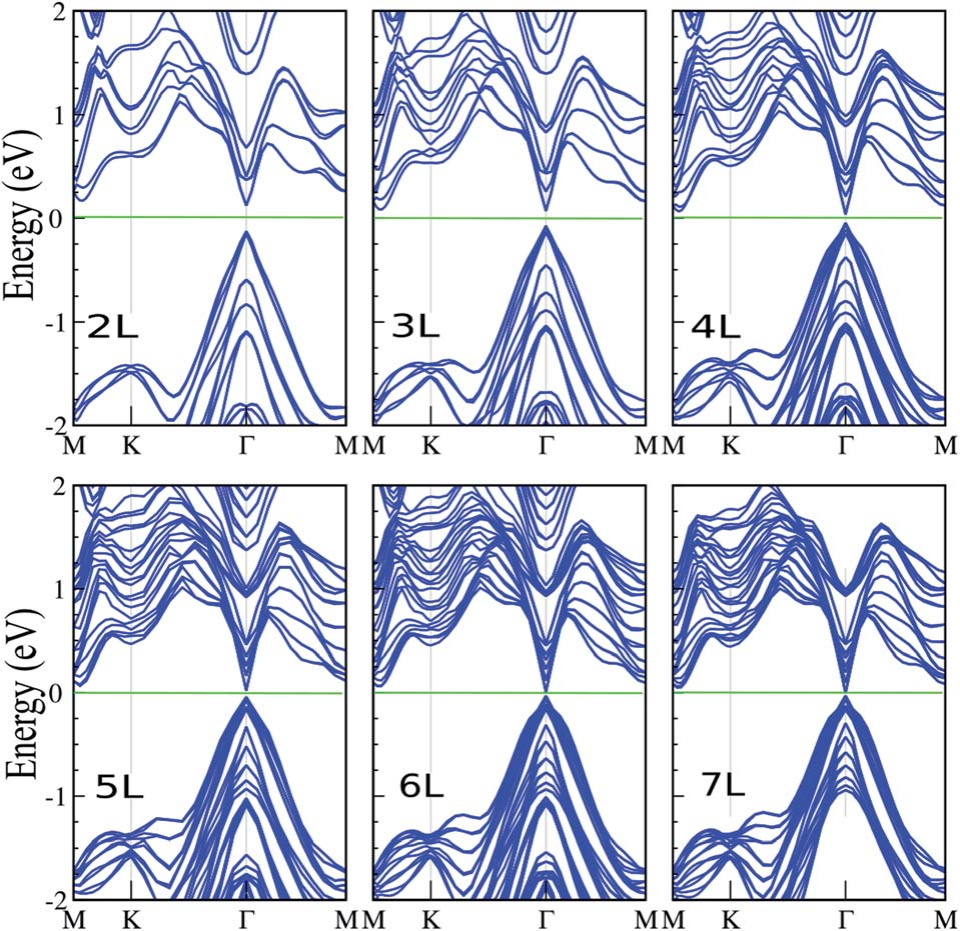
Electronic band structure of SiBi within PBE + SOC as a function of layer thickness with *L* = 1–7 layers. The zero energy is set to the Fermi level.

Here, we investigate the effect of layer thickness on the electronic properties of the SiBi lattice. Fig. 4 shows the electronic band structure of SiBi within PBE + SOC upon increasing the number of layers from two-layer (*L* = 2), to seven-layer (*L* = 7). For SiBi, the monolayer (*L* = 1) system shows a semiconductor with an indirect gap of 0.4 eV, while the VBM and CBM are located at the *Γ*-point and *M*–*K* direction, respectively. Our results show that the electronic band structure is strongly modified by the number of layers, and the semiconducting character is changed. Interestingly, a switching indirect-to-direct band gap occurs in the SiBi bilayer (*L* = 2) and the band gap decreases from 0.25 eV (*L* = 2) to 0.15 eV (*L* = 3). Transformation of the band structure from the monolayer to bulk of SiBi causes a strong modification at the CBM, while there is a change in the topology of the VBM upon an increasing number of layers. Our results show that the band gap decreased weakly upon increasing layer thickness. With an increased layer thickness of *L* = 4, 5 and 6 layers, the small band gaps of 85, 65 and 40 meV are obtained. We can see that the direct semiconducting charterer is preserved from *L* = 2–6 layers. As the number of layers increases from *L* = 2 to *L* = 6, the CBM moves from the *M*–*K* point toward the *Γ*-point, while the VBM stays at the *Γ*-point (independent of the slab thickness), thus direct band gaps are obtained at all layers. Interestingly, for the *L* = 7 layer, we found that a semiconductor-to-metal occurs. The variation of the band gap with number of layers can be explained by the effective reduction of the screening of the electrostatic interactions for few-layer systems, as well as quantum confinement effects of the electrons within the quasi-2D, finite thickness material.

## 5 Electric field

**Fig. 5:**
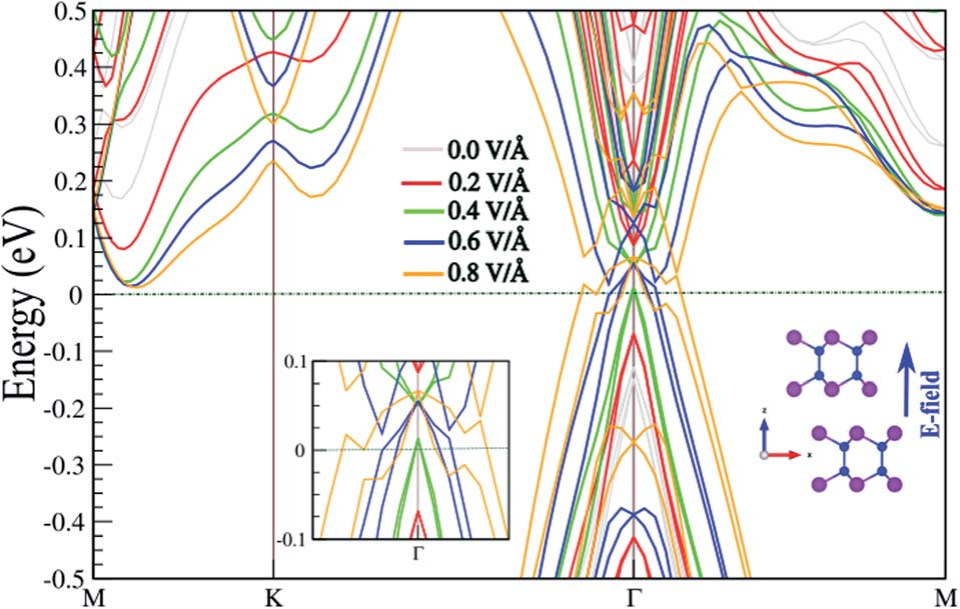
Electronic band structure of SnBi monolayer within PBE + SOC as a function of an E-field parallel to the *z*-axis, considering SOC. The optimized atomic structure the parallel electric field is applied to, and also the zoomed-in electronic structure, are shown in the insets. The zero energy is set to the Fermi level.

In the following, the effect of an E-field on the electronic properties is investigated. The existence of interlayer distance in the SiBi bilayer gives rise to a potential difference between the two atomic layers, which is potentially intrinsically useful in tuning the electronic properties by application of a perpendicular E-field. The electronic band structure of the SnBi bilayer, considering SOC as a function of the E-field parallel to the *z*-axis, is shown in Fig. 5. The corresponding optimized atomic structure for a perpendicular E-field varying from 0 to 0.8 V Å^−1^, and also a zoomed-in electronic structure illustration, are shown in the insets. Notice that the electronic structure is strongly modified with application of an E-field. The SiBi bilayer is a semiconductor with a direct band gap 0.4 eV in the absence of an E-field (0.0 V Å^−1^). When the E-field strength increases from 0.0 to 0.3 V Å^−1^, the band gap decreases from 0.25 to 0.15 eV. Interestingly, upon the critical value of 0.4 V Å^−1^, the band gap reaches zero, and we see that the VBM moves into the Fermi-level and a semiconductor-to-metal transition is occurs. As the electric field increases to greater than 0.4 V Å^−1^, the metallic characteristic is preserved. From the perspective of potential device applications, the ability to tune the electronic properties, *e.g.* by controlling the Fermi-level *via* an E-field, is highly desirable.

## 6 In-plane strain

**Fig. 6:**
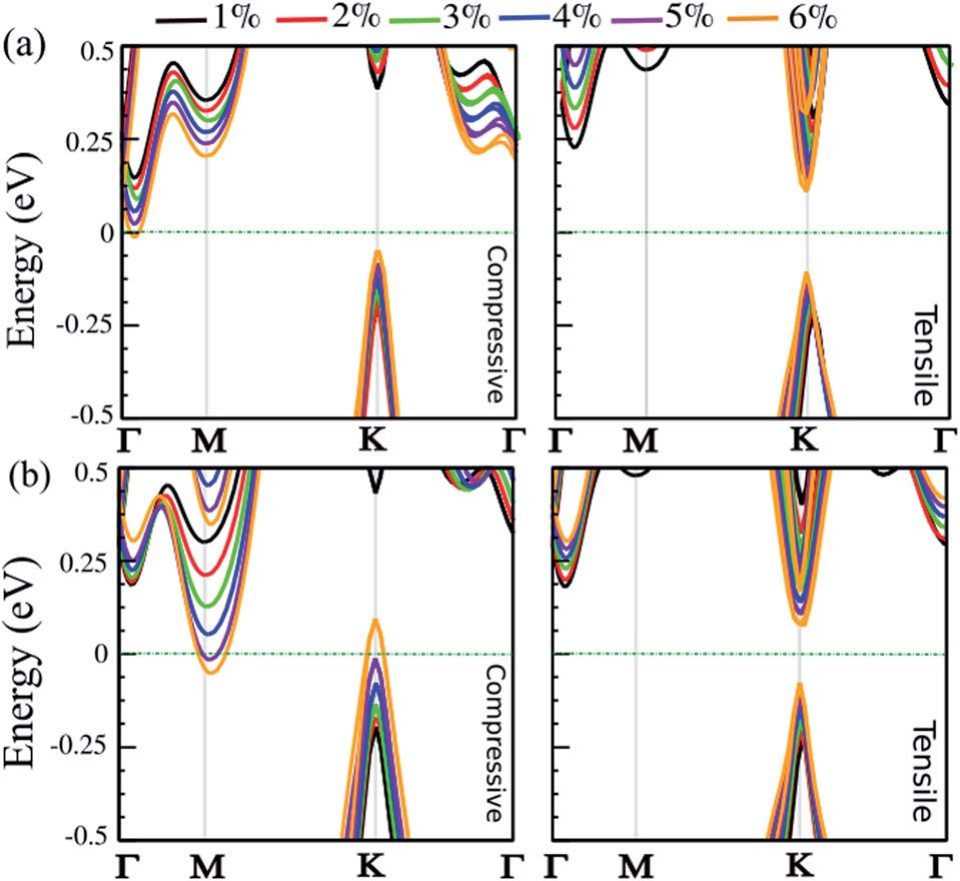
Electronic band structure of the SiBi monolayer within PBE + SOC as a function of (a) uniaxial and (b) biaxial strain, under tensile (right) and compressive (left) strain. The zero energy is set to the Fermi level.

Strain engineering is a robust approach to tune electronic properties and topological behavior. Here, we explore the effect of in-plane and out-of-plane strain in uniaxial and biaxial directions on the electronic properties of the SnBi monolayer. The strain is defined as *ε* = (*a* ± *a*_0_)/*a*_0_ × 100, where *a* and *a*_0_ are the strained and non-strained lattice constants, respectively. The positive and negative sign denotes tensile and compressive strain, respectively. The uniaxial strain is applied along the zigzag direction, while biaxial strain is applied along the *a*–*b* axis. The electronic band structure of the SiBi monolayer within PBE + SOC as a function of uniaxial and biaxial strain with SOC is shown in Fig. 6(a) and (b), respectively. When compressive uniaxial strain increase from 1% to 5%, the band gap decreases: 0.35 eV (1%), 0.3 eV (2%), 0.25 eV (3%), 0.2 eV (4%) and 0.1 eV (5%) (see left panel of Fig. 6(a)). Interestingly, for the compressive uniaxial strain of 6% and larger, the semiconductor to metal transition occurs. Under uniaxial strain, the VBM at the *k*-point, continuously shift upwards resulting in hole doping, while CBM around the *Γ*-point moves into the Fermi-level. For uniaxial tensile strain, we can see that the situation is different. For the tensile strain, with magnitude of 1%, the indirect band gap is 0.45 eV. Increasing the strain to 2%, the band gap increases to 0.5 eV, while the CBM will move from the *Γ*-point to the *K*-point in the BZ, hence we see an indirect-direct switching band gap. Under tensile strain larger than 2%, the direct band gap decrease from 0.4 (3%) to 0.2 eV (6%) and the semiconducting behavior is preserved. Notice that with increasing biaxial compressive strain, the band gap decreases to 0.4 eV (12%), 0.35 eV (2%), 0.25 eV (3%) and 0.15 eV (4%). Notice that the CBM will move from the *Γ*-point to the *M*-point for strains larger than 2%. For large strain (>4%), the VBM and CBM continuously touches the Fermi-level, leading the band gap to zero and the semiconductor transforms to a metal. Under a biaxial tensile strain, the indirect band gap is about 0.4 eV in the range 1–2%. The semiconducting characteristic remains for the strain larger than −2%. For large strain (>3%), the band gap decreases from 0.35 to 0.2 eV, in the range 3–6%, and an indirect-direct switching happens similar to the uniaxial situation. Notice that under large biaxial strain, the semiconductor remains for both tensile and compressive strain. These results reveal strain engineering dependent band gaps and electronic structure in the SiBi monolayer could be of use in high-performance nanoelectronic and optoelectronic devices.

## 7 Pressure

**Fig. 7:**
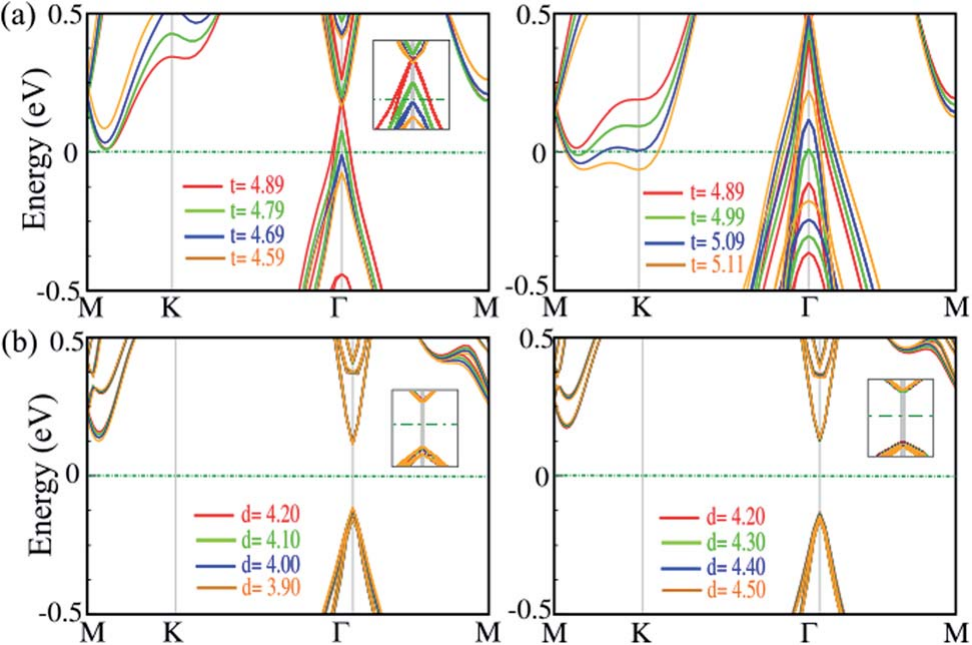
Electronic band structure of (a) the SiBi monolayer, and (b) SiBi bilayer, within the PBE + SOC as a function of interlayer distance under tensile (left) and compressive (right) strain. The zero energy is set to the Fermi level.

Strain along the vertical direction (out-of plane strain) *i.e.*, pressure, can change the interlayer distance and van der Waals interactions, which has been predicted to induce electronic state transitions. For the SiBi monolayer and bilayer, we investigate the effect of out-of-plane strain on the electronic properties. The electronic band structure of the SiBi monolayer as a function of thickness decrease (left) or increase (right) is shown in Fig. 7(a). In the SiBi monolayer, the thickness (distance between two Bi atoms) is 4.89 Å and has a band gap of 0.4 meV. Compared with the SiBi monolayer, when thickness decreases to 4.79 Å, the band gap decreases to 0.35 meV at the *Γ*-point, respectively. Our results show that the band gap decreases to 25 meV (at 4.69 Å) and reaches zero (at 4.59 Å) at the *Γ*-point. Increasing the thickness from 4.89 to 4.99 Å, the band gap decreases from 0.4 to 0.25 eV, respectively. When the thickness reaches 5.01 Å, a transition from semiconducting to metal occurs. Notice that for the larger thickness (from 5.01 to 5.29 Å) the metallic character is preserved. The electronic band structure of the SiBi bilayer as a function of interlayer distance decrease (left) or increase (right) is shown in Fig. 7(b). In the SiBi bilayer, the equilibrium interlayer distance is calculated to be 4.20 Å, while it is a semiconductor with a direct band gap of 0.25 eV. We found that with increasing interlayer distance from 4.20 to 4.30 Å, the direct band gap changed from 0.25 to 0.15 eV, respectively. For the interlayer distance of 4.30 Å, the CBM and VBM are located at the *Γ*-point; the reason being that the decreased or increased interlayer distance weakens the interlayer coupling, resulting in the SiBi bilayer keeping its individual electronic properties. Conversely, one can see that the band gap is constant when the interlayer distance decreases or increases (see Fig. 7(b)). Decreasing the distance from 4.20 to 3.90 Å, the structure remained a semiconductor with a direct band gap of about 0.25. Our results show that the electronic structure of the SiBi bilayer under increasing and decreasing interlayer distance, preserved its semiconducting character.

## 8 Conclusion

In summary, based on first-principles calculations, including fully relativistic effects, we investigated the influence of an electric field, layer thickness, and strain on the electronic structure of the SiBi nanosheet. Phonon spectrum and *ab initio* molecular dynamics simulation emphasizing the lattice and thermal stability of the studied monolayer. The results showed that the SiBi monolayer is a semiconductor material with a small indirect band gap of 0.65 eV. Interestingly, a stronger external electric field induces semiconductor-to-metal transitions in the SiBi bilayer. A similar trend has also been found under the effect of uniaxial and biaxial strains in the monolayer and bilayer of SiBi. Thus, the studied monolayer is very sensitive to both an external electric field and mechanical strain (in-plane and out-of-plane). Furthermore, the effect of layer thickness is studied and shows a tunable band gap as the number of layers increases. Overall, the SiBi monolayer is suggested to be a promising candidate for designing novel nanoelectronic devices.

## Conflicts of interest

The authors declare that there are no conflicts of interest regarding the publication of this paper.

## Supplementary Material
